# Toward a Universal Map of EEG: A Semantic, Low‐Dimensional Manifold for EEG Classification, Clustering, and Prognostication

**DOI:** 10.1002/ana.27260

**Published:** 2025-06-20

**Authors:** Laura Krumm, Dominik D. Kranz, Mustafa Halimeh, Alexander Nelde, Edilberto Amorim, Sahar Zafar, Jin Jing, Robert J. Thomas, M. Brandon Westover, Christian Meisel

**Affiliations:** ^1^ Computational Neurology, Department of Neurology and Berlin Institute of Health Charité – Universitätsmedizin Berlin Berlin Germany; ^2^ Bernstein Center for Computational Neuroscience Berlin Germany; ^3^ Charité – Universitätsmedizin Berlin, Einstein Center for Neurosciences Berlin Germany; ^4^ Institute for Theoretical Biology, Department of Biology Humboldt‐Universität zu Berlin Berlin Germany; ^5^ University of California, San Francisco (UCSF) San Francisco CA USA; ^6^ Massachusetts General Hospital Boston MA USA; ^7^ Harvard Medical School Boston MA USA; ^8^ Beth Israel Deaconess Medical Center Boston MA USA; ^9^ Center for Stroke Research Berlin Berlin Germany

## Abstract

**Objective:**

Prognostication in patients with disorders of consciousness (DOCs) remains challenging because of heterogeneous etiologies, pathophysiologies and, consequently, highly variable electroencephalograms (EEGs). Here, we use EEG patterns that are well‐characterizable to create a latent map that positions novel EEGs along a continuum. We asses this map as a generalizable tool to extract prognostically valuable information from long‐term EEG, by predicting outcome post‐cardiac arrest as a first use case.

**Methods:**

Categorizable EEGs across the health‐disease continuum (wake [W], sleep [rapid eye movement (REM), non‐REM (N1, N2, N3)], ictal‐interictal‐continuum [lateralized and generalized periodic discharges (LPD, GPD) and lateralized and generalized rhythmic delta activity (LRDA, GRDA)], seizures [SZ], burst suppression [BS]; 20,043 patients, 288,986 EEG segments) are arranged meaningfully in a low‐dimensional space via a deep neural network, resulting in a universal map of EEG (UM‐EEG). We assess prognostication after cardiac arrest (576 patients, recovery or death) based on long‐term EEGs represented as trajectories in this continuous embedding space.

**Results:**

Classification of out‐of‐sample EEG match state‐of‐the‐art artificial intelligence algorithms while extending it to the currently largest set of classes across the health‐disease continuum (mean area under the receiver‐operating‐characteristic curve [AUROCs] 1‐vs‐all classification: W, 0.94; REM, 0.92; N1, 0.85; N2, 0.91; N3, 0.98; GRDA, 0.97; LRDA, 0.97; SZ, 0.87; GPD, 0.99; LPD, 0.97; BS, 0.94). UM‐EEG enables outcome prediction after cardiac arrest with an AUROC of 0.86 and identifies interpretable factors governing prognosis such as the distance to healthy states over time.

**Interpretation:**

UM‐EEG presents a novel and physiologically meaningful representation to characterize brain states along the health‐disease continuum. It offers new opportunities for personalized, long‐term monitoring and prognostication. ANN NEUROL 2025;98:357–368

Despite the availability of electroencephalography (EEG) monitoring, prognostication for patients with disorders of consciousness (DOCs) remains challenging because of their diverse etiologies and underlying pathophysiologies.^1^ Current outcome prediction methods primarily depend on expert multimodal evaluations of clinical variables and EEG, and typically involve visual assessments, which can be time‐consuming and prone to subjective interpretation.^2^ Human visual interpretation may further be limited in appreciating complex long‐term data trends, transitions, or mixtures of states and may overlook subtle or not yet known features in EEG. Stimulation‐based approaches for prognostication may require complex setups not readily available everywhere.^3^, ^4^

Artificial intelligence (AI) has the potential to meet these challenges as it can, in principle, capture the full information contained in EEG. Previous AI models have focused on specific tasks, for example, distinguishing between normal and abnormal EEGs, detecting local and global slowing, or identifying epileptiform activity and SZ.^5^, ^6^, ^7^, ^8^, ^9^ However, these approaches often lack flexibility and generalizability, particularly when applied to data that deviates from their training sets. This limits especially the prognostication in patients with DOC, whose EEG patterns typically exists on a continuum and exhibit substantial variability, making traditional categorical classifications insufficient.^10^

Although EEGs of various neurological disorders (such as DOCs) are challenging to categorize, there exist distinct patterns where expert consensus is well established. Notable examples include the well‐characterized physiological transitions observed in wake–sleep or the ictal‐interictal continuum. These established patterns suggest that a low‐dimensional representation or embedding of EEG might suffice to capture much of its macroscale dynamics.^11^, ^12^, ^13^ We, therefore, propose a novel approach that leverages these clearly categorizable EEG patterns to generate a semantic, continuous embedding space that we then use for individualized prognostication in DOC. The idea is the following: we first use a large‐scale EEG dataset across the health‐disease continuum to create a universal map of EEG (UM‐EEG). This compact Euclidean space exhibits a low‐dimensional, semantic manifold in which the distances between EEGs are a measure of similarity. Second, we verify that this space enables state of the art classification and clustering of out‐of‐sample data of physiological as well as pathological states across our dataset. Third, we show that heterogeneous EEG patterns (such as those observed in DOC patients and which may not fall into 1 of the training classes) can be meaningfully positioned in the map because of its continuous nature. By projecting long‐term EEG recordings into this space over time, we are able to generate interpretable trajectories of how patients move in respect to the reference points of our map or in comparison to other patients. This approach also makes it possible to compare individual patients relative to their own baseline over time, providing insight into whether they maintain proximity to healthy or pathological patterns. The ability to capture such individual trajectories becomes particularly valuable in long‐term prognostication, where isolated assessments may miss important trends in patient status.

As a first application, we demonstrate how these trajectories along the manifold provide individualized outcome prognostication after cardiac arrest. The embedding offers immediate and interpretable insights into key prognostic factors, including the distance to healthy EEG dynamics, the variability of transitions between states, and time spent in burst suppression (BS) patterns. This approach leverages the prognostic value of continuous monitoring while providing a framework for more nuanced, patient‐specific analysis of neurophysiological recovery patterns.

## Materials and Methods

### Datasets

To create a UM‐EEG we used data from multiple sources covering a broad range of physiological and pathological states: a sleep dataset from 3,609 healthy subjects during polysomnography (PSG),^14^, ^15^, ^16^ a dataset covering the ictal‐interictal‐injury continuum (IIIC) with 1,557 subjects,^17^, ^18^ data from awake routine EEG recordings (2,355 subjects),^19^ and BS EEG data (20 subjects).^20^, ^21^ Furthermore, we use continuous EEG data from patients diagnosed with a disorder of consciousness following cardiac arrest (576 subjects) monitored over extended periods of time.^22^, ^23^, ^24^ Retrospective analysis of data for this project was conducted with waiver of informed consent under approved institutional review board protocols (BIDMC: 2022P000417; MGH: 2013P001024). For detailed information about data collection and specific inclusion criteria for each dataset, see Supplementary Methods in Data S1.

### Data Pre‐Processing

To ensure consistency across datasets, all recordings underwent standardized pre‐processing: resampling to 200Hz, notch filtering at 60Hz, bandpass filtering (0.5–40Hz), and re‐referencing to a common electrode configuration (F3‐C3, C3‐O1, F4‐C4, C4‐O2). We extracted non‐overlapping 10‐second segments from each recording and converted them to spectrograms using the multitaper method.^25^ For detailed information see Supplementary Methods in Data S1.

### Model Development

**FIGURE 1:**
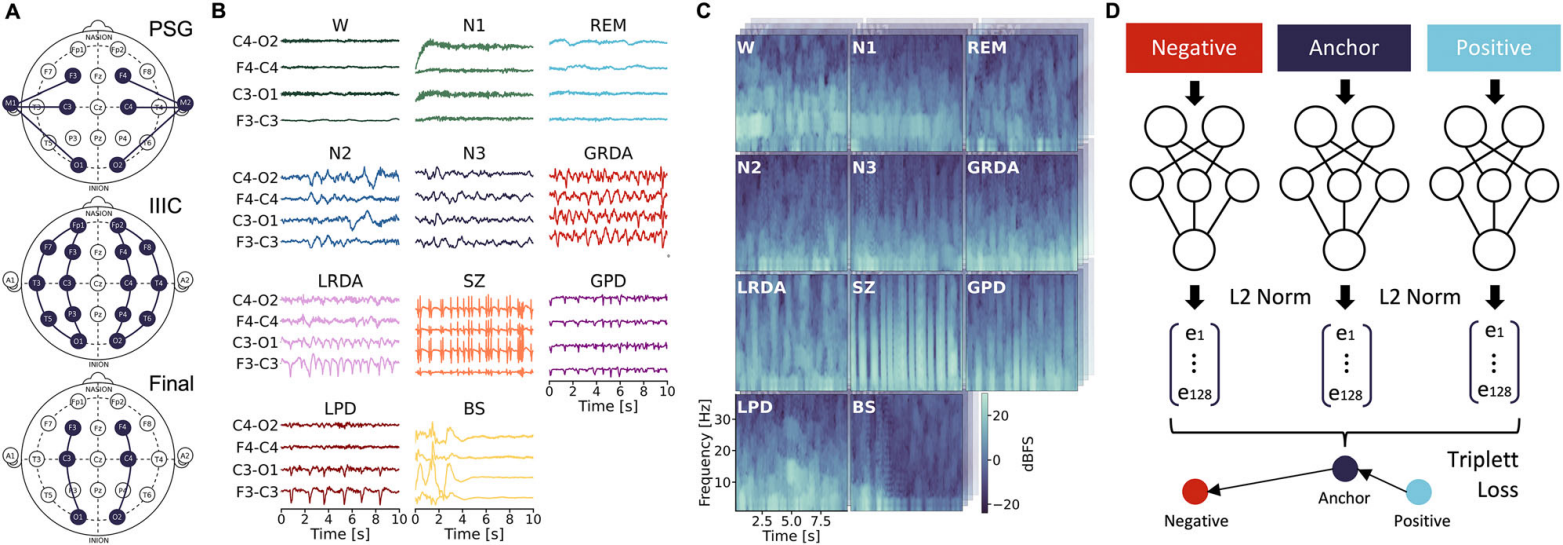
Data, pre‐processing, and deep learning model. (A) Electroencephalography (EEG) re‐referencing to include polysomnography (PSG) and ictal‐interictal‐injury continuum (IIIC) data. (B) Example segments for different activities (wake [W], rapid eye movement sleep [REM], non‐REM sleep stages N1–N3, seizures [SZ], generalized rhythmic delta activity [GRDA], lateralized rhythmic delta activity [LRDA], generalized periodic discharges [GPD], lateralized periodic discharges [LPD], and burst suppression [BS]. (C) Corresponding spectrograms, channel C4‐O2 in the front. (D) Schematic representation of the model and training in triplets. [Color figure can be viewed at www.annalsofneurology.org]

Generally, to ensure the model's robustness in a real‐world setting, throughout our study we rigorously validated on independent test datasets comprising EEG recordings from patients who were not included in the training set. This applies both to the initial division between training data (used for map generation) and test data (used for performance evaluation), as well as to the subsequent prognostication using a support vector machine (SVM). We used EEG spectrograms to develop a comprehensive latent space representation across physiological and pathological brain states. Segments (ie, spectrograms) were split into training and test sets at the patient level using an 80 of 20 ratio, ensuring that all segments from any given patient appeared only in either the training or test set. Both sets, therefore, contained a similar proportion of EEG segments for the different classes. To train a latent space EEG map, we adopted an approach based on triplet loss learning that has been successfully used in domains such as face recognition and clustering.^26^ The model maps data into a compact, 128‐dimensional (128D) Euclidean space, where the embeddings are projected onto a unit hypersphere, ensuring they lie within a confined space. A triplet loss function optimizes the network weights during training, by minimizing the distance between embeddings of the same class and maximizing the distance between embeddings of different classes (Fig 1D). More specifically, the network processes three 10‐second segments (each consisting of 4 spectrograms for the 4 EEG channels, respectively) simultaneously: an anchor (a segment from a certain class, eg, wake [W]), a positive (another segment from the same class, eg, W), and a negative (a segment from a different class, eg, N1). The model architecture, a Siamese inception network, shares weights across the 3 branches and updates them simultaneously. This design ensures that the learned embeddings accurately reflect the similarity between inputs, creating a space where distances correspond to similarity.^26^ To train the model with triplet loss, we instantiated a data generator that selects anchor, positive, and negative segment for each training batch. The generator randomly chooses 1 of the 11 training classes (W, N1, N2, N3, rapid eye movement [REM], seizure [SZ], generalized periodic discharges [GPD], lateralized periodic discharges [LPD], generalized rhythmic delta activity [GRDA], lateralized rhythmic delta activity [LRDA], and BS) as the anchor class. It, then, randomly selects an anchor segment and a positive segment from the chosen class. A negative class is selected at random from the remaining classes, and a segment from this class is fetched. This approach ensures that all class combinations are explored during training, promoting robust learning of class‐specific features. The batch‐based selection process enhances efficiency and ensures comprehensive coverage of the dataset.

### Performance Evaluation

To evaluate the model's performance, we monitored the loss function throughout the training process and made sure that the loss was not decreasing any longer before stopping. Additionally, we generated embeddings for a subset of the test data, consisting of 1,000 spectrograms per class, at specific epochs (epochs 0–5 in increments of 1, and epochs 10–50 in increments of 10) and trained a SVM using these test embeddings of the 11 classes (W, N1, N2, N3, REM, SZ, GPD, LPD, GRDA, LRDA, and BS) to monitor the F1 score for clustering of unseen data over time. After completing training, we saved the final model and generated embeddings for the complete test dataset. To evaluate the final model's ability to cluster unseen data, we used 5‐fold cross‐validation (at the patient level) using a SVM. Additionally, we computed the confusion matrix for all 5 folds and reported the mean and standard deviation for each entry. We also performed a binarized classification analysis by computing the area under the receiver‐operating‐characteristic curve (AUROC) and under the precision‐recall curve (AUPR) for each class against all others (1‐vs‐all). For each fold, we applied bootstrapping (50 iterations per fold) on the test‐fold data to calculate the mean ROC, PR curves, respective AUC values, and their 95% confidence intervals (CI).

**FIGURE 2:**
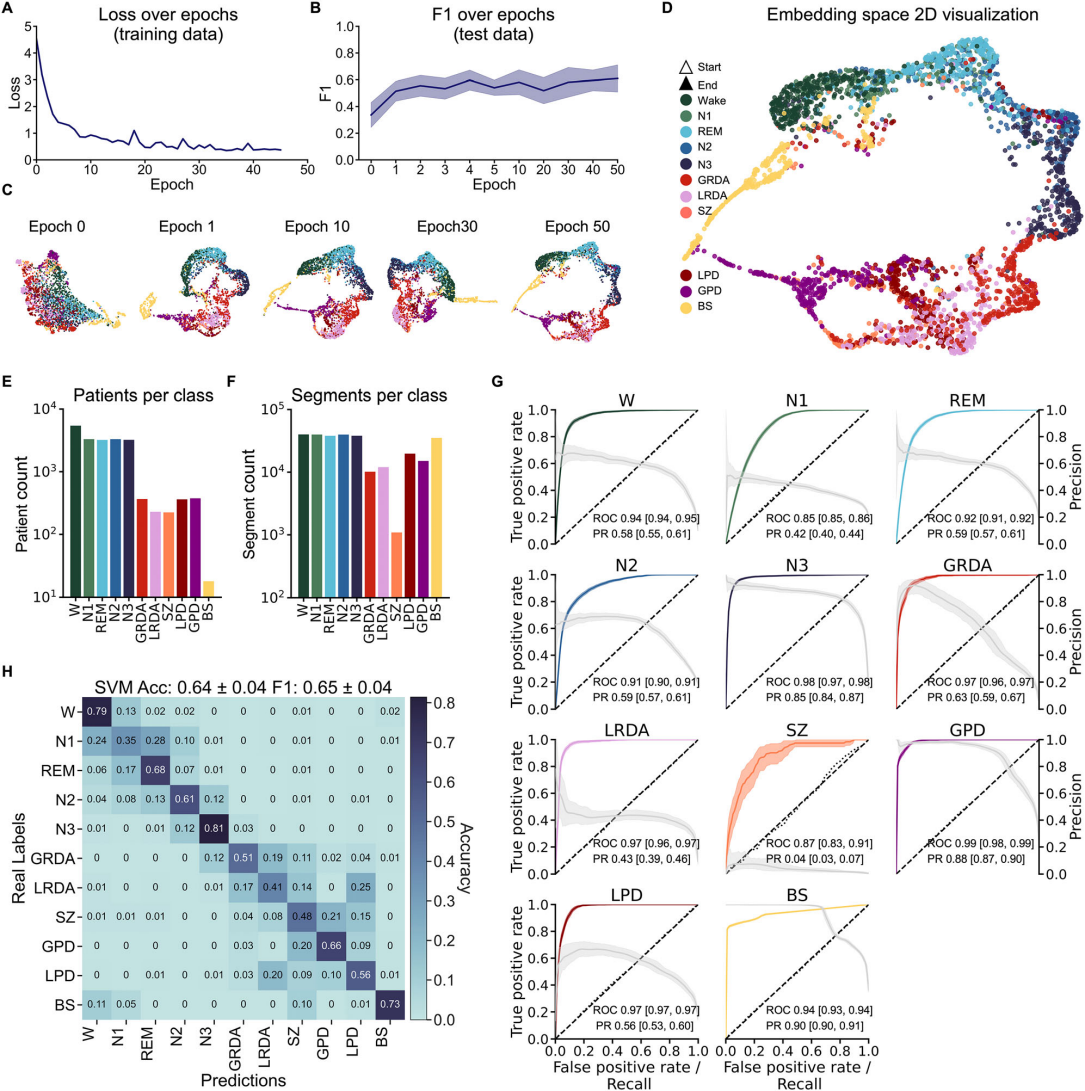
Training and state classification using the embedding space. (A) Loss over epochs during training. (B) For selected epochs, the same 1,000 test segments per class were passed through the model and classification performance was monitored with F1 scores (support vector machine [SVM], 5‐fold cross‐validation). (C) Embedding space of 300 test data points in 2‐dimensional (2D) representation (dimensionality reduction via uniform manifold approximation and projection [UMAP]) for the selected epochs. (D) Final embedding space representation in 2D via UMAP. (E) Number of patients per class for all data (80% training, 20% testing data). (F) Number of 10‐second segments per class for all data. (G) Receiver‐operating‐characteristic (ROC) and precision‐recall (PR) curves for a SVM trained on embeddings from the test set via 5‐fold cross‐validation. For each fold bootstrapping (50 times) was performed on the test data to compute the mean ROC/PR curves and their respective AUCs, as well as their 95% confidence intervals. (H) Confusion matrix for the mean classification accuracy between the test embeddings of the different classes using a SVM. [Color figure can be viewed at www.annalsofneurology.org]

To assess the semantic nature of the 128D embedding space, we used multidimensional scaling (MDS), a statistical technique that visualizes the level of similarity or dissimilarity between a set of objects in a lower‐dimensional space. We first calculated the median embeddings for each class in the 128D embedding space and then computed a distance matrix using cosine distances between all class medians. We applied MDS to this distance matrix to reduce the dimensionality to 2‐dimensional (2D), allowing us to visualize the relative positions of the class medians in a 2D space (Fig 2). To better comprehend the spatial distribution of our BS data in 128D space, we calculated the burst suppression ratio (BSR) for each BS segment. The BSR, also known as the burst suppression index (BSI), was computed using the following parameters: maximum amplitude of suppression of 10 microvolts, minimum duration of suppression of 0.5 seconds, and minimum duration of bursts of 0.2 seconds. We, then, investigated the spatial arrangement of BS patterns within the map across 3 categories: high proportion of suppression periods (BSR >0.7), intermediate proportion of suppression and burst periods (0.35 ≤ BSR ≤ 0.65), and high proportion of burst periods (BSR <0.3).

### Prognostication of Patient Outcomes from Trajectories in Embedding Space

We projected continuous data from post‐cardiac arrest comatose patients into the UM‐EEG to obtain trajectories in latent space. Specifically, for each patient, we processed consecutive 10‐second EEG segments through the map, generating a time series of embedding vectors. To assess the predictive power of these embedding trajectories for patient outcomes, we first projected the EEG into either 1 of the 11 classes (W, N1, N2, N3, REM, SZ, GPD, LPD, GRDA, LRDA, and BS) by calculating cosine distances from each time point to the median coordinate of each of the 11 classes. We assigned each segment to the nearest class to analyze how patients with different outcomes traversed the embedding space. Next, we quantified for each patient the relative time spent in each of the 11 classes. We used 5‐fold cross‐validation using a SVM to classify patients according to their outcomes (Cerebral Performance Category (CPC) 1 and 2 [ie, recovery] vs CPC 5 [ie, death]). We report performance in terms of AUROC and the corresponding 95% CI.

Recognizing that our map was trained on specific EEG patterns and that the cardiac arrest dataset might present a spectrum of patterns not directly aligning with the predefined classes used in training, we also conducted a more granular analysis of patient trajectories. Specifically, we generated an evenly spaced grid on the unit hypersphere by randomly sampling up to 100,000 data points in 128 dimensions, projecting them onto the hypersphere, and applying repulsive forces to distribute them evenly across the surface. Each point was then assigned a reference number. We computed the cosine distance from each patient data point to the nearest grid point rather than the nearest class median. This approach allowed for a finer resolution of the trajectories within the continuum embedding space, providing a detailed mapping of patient states without strict reliance on the predefined classes.

### Dynamics of Trajectories and Linear Discriminant Analysis

To assess the dynamical information contained in the trajectories in our map, we calculated 45 parameters from classical time series analysis (Table). These parameters included temporal and symbolic dynamics as well as complexity measures derived from the time series of classes (W, N1, N2, N3, REM, SZ, GPD, LPD, GRDA, LRDA, and BS), as well as spatial measures derived directly from the 128D embeddings. We derived these parameters from the time series of classes in the cardiac arrest dataset. We investigated which combinations of 2 or 3 parameters best separated the 2 outcome groups (CPC 1 and 2 vs CPC 5) by performing a linear discriminant analysis (LDA) for each combination of parameters. This approach demonstrates the utility of the embedding to derive explainable and intuitively understandable dynamics parameters that adequately predict neurologic outcome.

## Results

### Building a UM‐EEG

We included EEGs from a wide range of physiological and pathophysiological states, including the healthy/normal brain activity continuum (W, N1, N2, N3, and REM), the ictal‐interictal‐injury continuum (GPD, LPD, GRDA, LRDA, and SZ) and BS, to create a comprehensive latent space map of EEG (Fig 1). Systematic initial evaluations on the IIIC dataset indicated that performance increased strongly when more than 1 channel was used and then reached some stable plateau around 4 channels with only a minor performance increase when all 16 channels were used (Fig S1). We, therefore, re‐referenced all data to 4 bipolar channel pairs to include data from all datasets (Fig 1A). Model performance monitored with loss function and F1 score indicated stable performance improvement during training without signs of overfitting (Fig 2A,B). For visualization purposes, the 128D embedding test data was projected onto a 2D space using uniform manifold approximation and projection (UMAP).^27^ As training progressed, the classes exhibited increased clustering, ultimately resulting in a complex embedding space with some classes disjunct from and others merging into each other (see Fig 2D). Notably, already in the 2D projection, semantic clustering emerged, including distinct and meaningful spatial arrangements capturing, for example, natural state transitions during sleep (W → N1 → REM → N2 → N3).

### Classification of Unseen EEG in UM‐EEG

We first assessed classification of EEGs in the 128D embedding space using the receiver‐operating‐characteristic (ROC) and precision‐recall (PR) curves for all classes in a binarized classification approach (1‐vs‐all) (see Fig 2G). The mean AUROC curve and corresponding 95% CI for each class indicated robust class separation: W (0.94 [0.94–0.95]), REM (0.92 [0.91–0.92]), N1 (0.85 [0.85–0.86]), N2 (0.91 [0.90–0.91]), N3 (0.98 [0.97–0.98]), GRDA (0.97 [0.96–0.97]), LRDA (0.97 [0.96–0.97]), SZ (0.87 [0.83–0.91]), GPD (0.99 [0.98–0.90]), LPD (0.97 [0.97–0.97]), and BS (0.94 [0.93–0.94]). For the IIIC (SZ, LPD, GPD, LRDA, and GRDA), apart from SZ, these results match or exceed discrimination to recently published results,^17^ while including more classes. The relatively lower performance for SZ may be attributed to the limited number of segments available for this class and the high variability of patterns within this category. Similarly, AUPR curves showed effective binary classification based on the latent space map (see Fig 2G). Across all 11 classes, the model demonstrated effective learning in clustering achieving a final F1 score of 0.65 ± 0.04 (compared to 0.08 ± 0.01 for a random predictor) and an accuracy of 0.64 ± 0.04 (compared to 0.09 ± 0.01 for a random predictor for multiclass classification). Figure 2H shows the confusion matrix across all individual classes. Using the Youden Index as the single operational point determined during training provided the following sensitivity and specificity values for each class in the test data; sensitivity: W (0.90 [0.84–0.96]), REM (0.92 [0.88–0.95]), N1 (0.94 [0.90–0.97]), N2 (0.88 [0.83–0.92]), N3 (0.94 [0.90–0.97]), GRDA (0.78 [0.73–0.84]), LRDA (0.86 [0.81–0.90]), SZ (0.75 [0.60–0.89]), GPD (0.90 [0.85–0.94]), LPD (0.88 [0.83–0.93)]), and BS (0.79 [0.75, –0.82]); specificity: W (0.89 [0.88–0.91]), REM (0.83 [0.81–0.85]), N1 (0.74 [0.72–0.76]), N2 (0.83 [0.80–0.85]), N3 (0.93 [0.92–0.94]), GRDA (0.89 [0.87–0.90]), LRDA (0.88 [0.87–0.89]), SZ (0.86 [0.85–0.88]), GPD (0.94 [0.93–0.95]), LPD (0.86 [0.85–0.88)]), and BS (0.99 [0.98–0.99]). Collectively, these results demonstrate effective classification of out‐of‐sample data based on distinct clustering in latent space. This suggests a highly semantic embedding space of the various EEG states and patterns.

### Semantic Representation across the Health‐Disease Continuum in UM‐EEG

**FIGURE 3:**
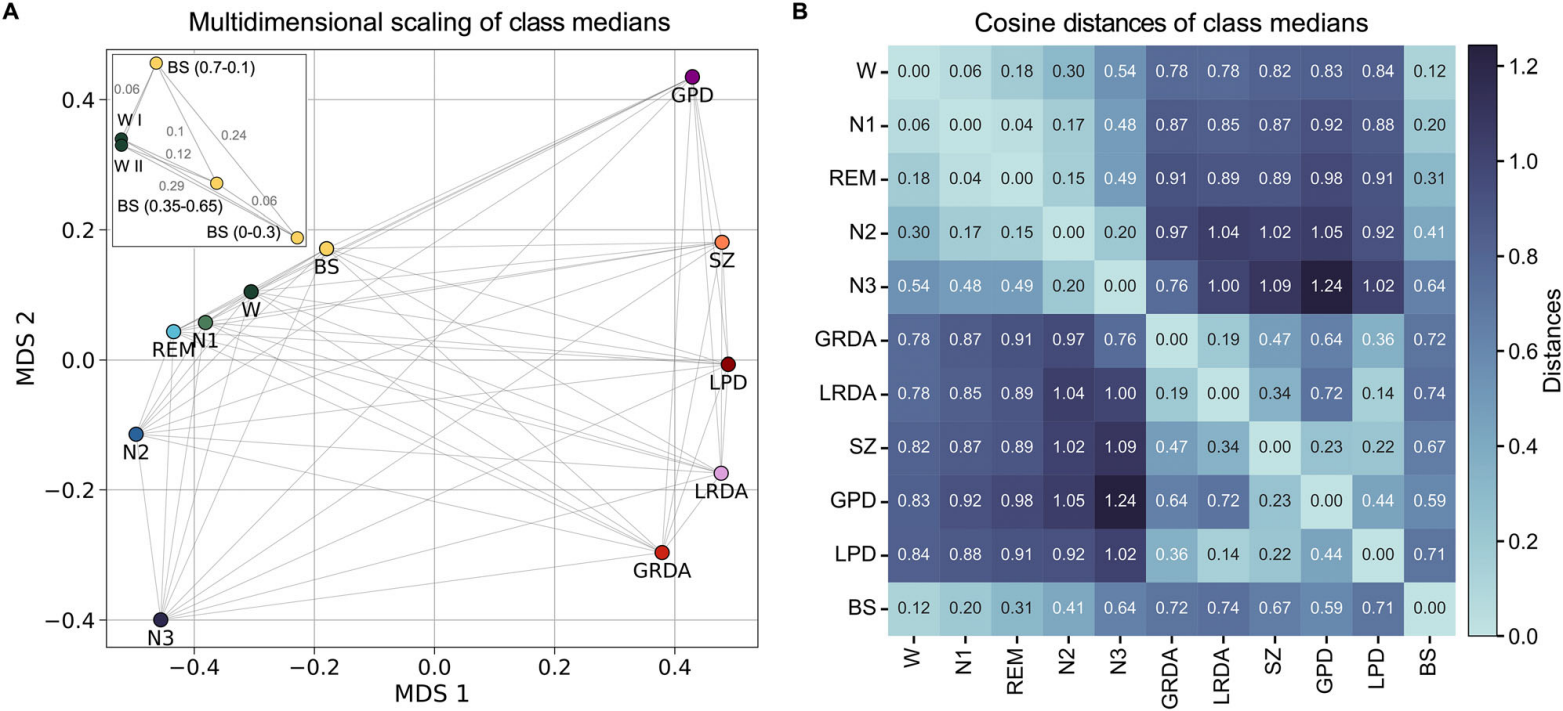
Semantic representation of electroencephalography (EEG) states across the health‐disease continuum in embedding space. (A) Class medians and their respective cosine distances are projected into 2‐dimensional (2D) using multidimensional scaling, which preserves the spatial arrangement in 128D as closely as possible. The inlay provides a more nuanced distinction within the burst suppression and wake classes. Our wake resting state data originates from 2 distinct datasets (WI from the HSP and WII from the HED dataset). For the burst suppression data, we applied an additional grouping based on the burst suppression ratio (BSR). This grouping resulted in 3 categories: high proportion of suppression periods (BSR >0.7), intermediate proportion of suppression and burst periods (0.35 ≤ BSR ≤0.65), and high proportion of burst periods (BSR <0.3). (B) Matrix representation of the cosine distances of all class medians in 128D. [Color figure can be viewed at www.annalsofneurology.org]

In a semantic latent space, similar data points are positioned closer together, reflecting their semantic (meaningful) relationships. To determine semantic properties of the EEG map beyond the 2D UMAP visualization, we next analyzed the 128D embedding space. Specifically, we computed the median vectors for all classes in this space and calculated the cosine distances between class medians. Additionally, MDS was used to create a 2D representation that preserves the inter‐class distances from the 128D space as closely as possible (Fig 3A). For a precise comparison, we also presented all pairwise distances in a matrix format (see Fig 3B). Results from this analysis revealed a meaningful spatial arrangement in the 128D space, consistent with MDS and visual assessment of the 2D UMAP projection.

First, healthy and pathological states appeared to be clearly separated. The pathological domain (GPD, SZ, LPD, LRDA, and GRDA) was far from physiological states, with generalized rhythmic and periodic patterns (GPD, GRDA) flanking the respective lateralized analogues (LPD, LRDA). Second, arrangement within the physiological domain closely reflected the natural state transitions of sleep stages within sleep cycles (W → [N1, REM] → N2 → N3). Third, the model captured nuanced differences within the BS category and its relationship to normal W patterns. Specifically, in a subanalysis, we computed the mean BSR.

Across all channels from BS segments to quantify the proportion of burst and suppression patterns more precisely. We observed that EEG segments with BSR <0.3 (high proportion of suppression) were more distant from W (cosine distance = 0.29). Patterns with BSR between 0.35 and 0.65 showed an intermediate distance to W (cosine distance = 0.12), whereas patterns with BSR between 0.7 and 1 (burst‐dominant) were closest to W (cosine distance = 0.06) (see Fig 3A inset). These results indicate that there is a gradient in the embedding space, where BS patterns with a higher proportion of bursts are arranged closer to the W state, and, conversely, BS patterns with higher proportion of suppression are arranged further away from W. Figure S2 provides detailed examples how ambiguous or mixed segments are positioned along a continuum space in the map that accurately reflects the BSR gradient. Fourth, the model correctly assigned W EEG from different datasets to the same place in the map. As our W data class was comprised from 2 distinct datasets (HSP W data and HED resting state), we projected both W test data sources (W I and W II) separately into the map in a subanalysis (see Fig 3A inset). We found the class medians for these 2 to be in very close proximity (distance = 0), indicating no significant differences between the datasets, and that, consequently, distances between states could not simply be attributed to originating from different datasets.

### Patient Trajectories in UM‐EEG Predict Outcome after Cardiac Arrest

**FIGURE 4:**
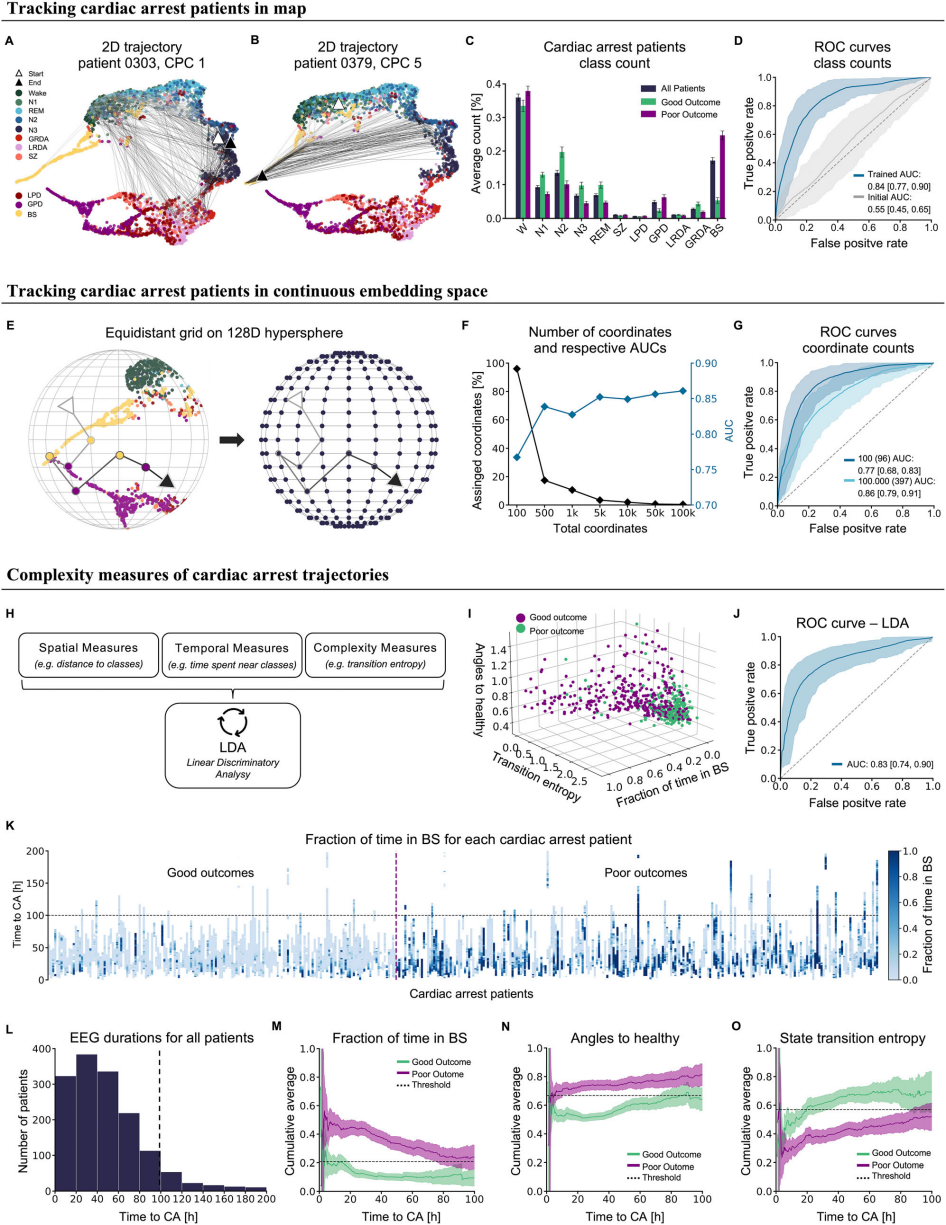
Patient trajectories in universal map of electroencephalography (UM‐EEG) predict outcome after cardiac arrest. (A) Two‐dimensional (2D) uniform manifold approximation and projection [UMAP] representation (of 128D hypersphere) for a cardiac arrest patient trajectory in with a good outcome (CPC 1) projected into the map over time. The white triangle indicates the starting point and the back triangle the end of the trajectory. (B) Example trajectory of a cardiac arrest patient with a poor outcome (CPC 5). (C) Average time spent in each class according to outcome. Each trajectory segment was assigned to the class with the smallest cosine distance. The class counts for all patients were normalized by their total number of segments and averaged. The error bars indicate the standard error of the mean. (D) Receiver‐operating‐characteristic (ROC) curves for classification of good (CPC 1,2) versus poor (CPC 5) outcome based on class counts for each patient in trained (*blue*) versus untrained embedding space (*gray*). (E) Generation of an equidistant grid (coordinates) on the 128D hypersphere. Instead of tracking cardiac arrest patients in terms of closest classes, they were assigned to the closest coordinates for finer resolution. (F) Performance as a function of total size of coordinate system and the percentage of closest coordinates. (G) ROC curves for cardiac arrest outcome prediction on grids of 100 and 100,000 coordinates. (H) Schematic overview of metrics used for time series analysis of trajectories. (I) Visualization of separation of outcome achieved with 3 best linear discriminant analysis (LDA) parameters (time spent in burst suppression, distance to healthy, and state transition entropy). (J) Corresponding ROC curve. (K) Individual patient trajectories according to time spent in burst suppression. (L) Histogram of durations of EEG data. We chose a cut‐off after 100 hours because of insufficient data. (M,N,O) LDA parameters provide intuitive markers predictive of prognosis after cardiac arrest. Dashed horizontal lines indicate the center between the mean of patients with good outcomes and the mean of patients with poor outcomes. [Color figure can be viewed at www.annalsofneurology.org]

We hypothesized that projections of long‐term EEGs into the semantic embedding would allow for effective characterization of these trajectories relevant for outcome prediction and identification of important predictive data signatures. We generated patient trajectories from 576 patients after cardiac arrest by feeding consecutive 10‐second segments into the model, mapping each patient's EEG evolution over time in embedding space (Fig 4A,B).

We first assessed the prognostic information content contained in map trajectories by assigning each 10‐second segment to the 11 training classes according to the smallest cosine distance to class medians (see Fig 4A,B). The relative time spent in different classes differed between patients with good (CP 1 and 2) and poor outcomes (CPC 5) (see Fig 4C). Patients with good outcomes spent more time in states close to healthy conditions (W, N1, N2, N3, and REM) compared to those with poor outcomes (*p* = 1.99e−16, Mann–Whitney *U* test). Conversely, patients with poor outcomes spent more time in pathological states (SZ, LPD, GPD, LRDA, and GRDA; *p* = 2.29e−02) and particularly in BS (*p* = 1.47e−27). Using the information of relative time spent in these classes provided good outcome prediction after cardiac arrest with an AUROC of 0.84 [95% CI, 0.77–0.90] and a sensitivity of 0.85 [95% CI, 0.75–0.94] and specificity of 0.78 [95% CI, 0.70–0.87] (see Fig 4D). In comparison, projections into an untrained map yielded a significantly worse AUROC of only 0.55 [95% CI, 0.45–0.65] (*P* = 2.19e−224, sensitivity: 0.44 [95% CI, 0.32–0.57], specificity: 0.76 [95% CI, 0.68–0.84]), demonstrating that the embedding space, trained on both healthy and pathological classes, enhanced the meaningfulness and prognostic value of cardiac arrest EEGs.

Assigning segments to the nearest training class provided already good prediction performance result, but effectively transformed the problem into a classification task limited by the number of classes the embedding was trained on. Given the highly semantic nature of the embedding space and the heterogeneity of cardiac arrest EEG patterns, we hypothesized that outcome prediction would improve further when trajectories were not limited to predefined classes, but could make use of the full continuum spanned by our map. We, therefore, applied a more nuanced approach that leveraged the continuous nature of the embedding space by projecting an evenly spaced 128D grid onto this sphere to serve as a coordinate system (Fig 4E). Instead of assigning each segment merely to the closest class, we assigned it to the closest coordinate. As we increased the number of coordinates, making the grid more fine‐grained, the AUROC increased leading to an AUROC of 0.86 [95% CI, 0.79–0.91] (sensitivity: 0.87 [95% CI, 0.83–0.91], specificity: 0.80 [95% CI, 0.70–0.83]) for the most fine‐grained coordinate system with 100,000 coordinates (from 0.77 [95% CI, 0.68–0.83] (sensitivity: 0.76 [95% CI, 0.65–0.88], specificity: 0.68 [95% CI, 0.55–0.81]) with 100 coordinates, *p* = 4.38e−72). This continuum‐based approach outperformed prognostication based on traditional classes (*p* = 1.40e−07) (see Fig 4G). Although increasing the number of coordinates improved the AUROC, the number of coordinates actually used increased only slightly (96 coordinates used for a grid size 100, 397 for grid size 100,000) (see Fig 4F). This indicates that the AUROC improved because of a finer grid representation of the cardiac arrest data clustered in distinct areas across the hypersphere. To mimic real‐world application where this method is applied to a new clinical center, we split the folds not only along patients, but also along the 5 individual clinical centers. This ensures that each of the 5 centers constitutes an independent, out‐of‐sample validation data set. This analysis provided again a better prediction using the coordinate counts (mean AUROC 0.83 [95% CI, 0.70–0.97], sensitivity: 0.81 [95% CI: 0.70–0.94], specificity: 0.80 [95% CI, 0.67–0.93]) in comparison to the class counts (mean AUROC 0.80 [95% CI, 0.68–0.93], sensitivity: 0.83 [95% CI, 0.69–0.97], specificity: 0.76 [95% CI, 0.67–0.85]) (Fig S3).

Finally, we determined what dynamical characteristics of the trajectories in embedding space, aside from the relative time spent in certain states, were predictive of outcome. For this purpose, we derived well established spatial, temporal, and complexity parameters from the time series of classes, as well as the trajectories directly (Table S1) and applied LDA to identify parameters that separated “poor” and “good” outcomes (see Fig 4H). The best discrimination was achieved with 3 parameters, indicating that a low distance (angle) to the healthy continuum together with a low amount of time in BS and a high state transition entropy were predictive of a good outcome. Although using only these 3 parameters provided a comparable AUROC (0.83 [95% CI, 0.74–0.90]) (see Fig 4I,J), the simplicity of these parameters exemplifies the predictive, meaningful and understandable information gained from the map that can be used for monitoring patients. Specifically, these metrics match the intuitive assumption that a healthy brain displays complex patterns in state transitions, is closer to the healthy states (W and sleep) and spends little to no time in BS. Meaningful separation in these metrics was already evident after the first 12 hours after cardiac arrest (see Fig 4M–O) indicating that these metrics can be used to benchmark a patient to themselves over time and, potentially, also in other, heterogeneous diseases and patient cohorts beyond cardiac arrest.

## Discussion

We present a latent space embedding for EEG that covers the health‐disease continuum and preserves the physiologic ordering within brain states. Our findings indicate that various brain states, physiological and pathological ones, can be represented and understood as points or trajectories within this low‐dimensional latent space, offering new applications in diagnosis, prognosis, and data augmentation for automated classification.

To generate our UM‐EEG, we projected the training data onto a confined space (a 128D hypersphere) while allowing the data to arrange itself meaningfully. Perhaps surprisingly, we found that data from the IIIC can be accurately represented in our embedding space despite a lower channel count (4 channels instead of 16). This enabled the creation of a unified space that incorporates diverse datasets, including polysomnography data where only a low channel count is typically available. The resulting embedding space not only organizes the training classes in a semantically significant manner, but also enables a continuum between different classes. This continuum reflects, for example, the natural progression of sleep stages (W → [N1, REM] → N2 → N3), suggesting that the map captures a lower‐dimensional manifold of sleep stage transitions within this higher‐dimensional space. Moreover, pathological states are arranged much further away from W than other healthy states (sleep stages). The continuous and semantic nature of this embedding space offers an additional advantage: it allows for the meaningful projection of unseen data that may not precisely match the training classes. New data self‐arranges within the space in semantically appropriate positions, demonstrating flexibility with novel inputs. Our subanalyses on sleep stage transitions and BS patterns along with the model's effectiveness to predict outcomes using high‐resolution reference grid points, therefore, provide empirical evidence that also ambiguous or mixed EEG patterns are meaningfully positioned in the map. Our approach, therefore, not only demonstrates the potential for complex data relationships to be represented in a more interpretable form, but also provides a framework for categorizing unseen data.

Although the model is not a classifier per design, classification naturally emerges from our embedding space as a spatial clustering problem that can be solved using standard clustering algorithms such as SVMs. Framing classification as a clustering problem enables us to easily classify both physiological and pathological EEG patterns, which exceed current AI approaches that are mostly limited to only a few pathological classes.^5^, ^6^ Therefore, direct comparison of our study to existing research is challenging. For classes derived from the IIIC dataset, our method matches or exceeds the state of the art, for 4 of 5 AUROC and 2 of 5 AUPR scores (AUROC comparison of our results with state of the art: SZ 0.87 vs 0.92, LPD 0.97 vs 0.96, GPD 0.99 vs 0.93, LRDA 0.97 vs 0.94, GRDA 0.97 vs 0.80 and AUPR comparison: SZ 0.04 vs 0.78, LPD 0.56 vs 0.91, GPD 0.88 vs 0.75, LRDA 0.43 vs 0.70, GRDA 0.63 vs 0.48).^17^ Importantly, although a classification approach yields valuable insights, it may not adequately capture the full complexity of EEG patterns. In this study, we showed that states often exist on a continuum, so reducing analysis to discrete classification results in some loss of information.

As a first use case, we projected data from cardiac arrest patients into the embedding space to predict outcome. Our approach (AUROC 0.86 [95% CI, 0.79–0.91]) surpassed existing stimulation‐based tests in predicting outcomes for cardiac arrest patients using only resting‐state EEG data (AUROC 0.70 ± 0.04).^3^ Our prognostication results improved further when using an approach that captured more continuous information compared to a classification approach (AUROC of our grid compared to map classification). Although our predictive performance was slightly lower than a specifically trained classifier for the same dataset (AUROC, 0.88),^28^ the strength of our approach lies in its flexibility and generalizability. Unlike cohort‐specific methods, which are common in machine learning, we suggest that our approach can handle patients with additional diseases or atypical cardiac arrest progressions. Moreover, our method is applicable across different etiologies without the need for retraining the model. As we predict outcomes based on a reduced number of interpretable measures such as distance to healthy‐state, time spent in BS, and state transition entropy, our approach still provides good prognostic value (AUROC, 0.83) while offering generalizability and interpretability. We provide meaningful scores for clinicians that could be monitored over time and aid in decision‐making. Providing interpretable scores also offers the opportunity for individualized assessments. Specifically, we demonstrate capability to generate personalized trajectories for each patient within the embedding space that can be transformed into meaningful information by applying time series metrics that would not be applicable to raw EEG. This approach, therefore, allows patients to be benchmarked against their own baselines over time rather than against other patients in the same cohort, as is commonly practiced in machine learning.

Further use cases of UM‐EEG might include clustering patients to relate them to similar individuals with existing medical reports, thereby aiding clinicians in their diagnostic processes and EEG reading. Additionally, it could enable the generation of new reports based on those of spatially close patients within our embedding space using large language models. Beyond this, our UM‐EEG can have several meaningful clinical applications. Even in countries with advanced healthcare systems, many EEGs are interpreted by physicians who lack specialized fellowship training in this area, and which also contributes to misinterpretation of EEGs.^29^, ^30^ Furthermore, the growing number of EEG referrals has created a significant workload, even for specialized centers, a problem that is particularly exacerbated during interpretation of long‐term EEGs, including prognostication. In current clinical practice, appreciation of all the complex information contained in days‐ and week‐long EEGs is challenging. Projection of such complex EEG trajectories into the embedding space as an automated, end‐to‐end pipeline along with up‐to‐date outcome prediction scores (eg, in a bedside dashboard) could be valuable in that sense as it may condense the information into a dense, digestible format for integration into clinical workflow and decision support for clinicians. We hope to contribute to enhancing patient care by providing real‐time, data‐driven insights to healthcare professionals, potentially improving treatment efficacy. To work toward this goal we will continue training, expanding, and validating UM‐EEG. Although training of UM‐EEG required significant GPU resources (approximately 28 hours training on a V100 GPU), inference and generation of embedding space was fast (approximately 2 second on a conventional CPU). Therefore, real‐time application without the need of dedicated hardware appears feasible in clinical settings.

Although our approach demonstrates promising results, it is important to acknowledge its limitations. Our current use of only 4 EEG channels may result in the loss of important information, particularly for localized patterns such as activities from SZ and the IIIC (LRDA and LDA). However, our subanalysis revealed that we still achieve good classification performance with this limited channel count compared to a higher channel count of 16 electrodes, suggesting the robustness of our method. Future work could address this constraint through transformer architectures or masked variational autoencoders capable of handling datasets with variable numbers of channels. As the model is trained in a supervised manner, another consideration is the potential for inaccuracies in our labelled segments (given they were labelled by humans). However, our embedding space is a continuum, which allows for more flexible placement of ambiguous or potentially mislabeled segments compared to a traditional classifier. Another limitation could be that our EEG segments are converted into spectrograms and future work will determine the utility of other data representations, including raw voltages or statistical data features. Finally, although our model is trained primarily to learn the (dis‐)similarity between EEG patterns, making it, therefore, less dependent on class labeling, human mislabeling of classes may still potentially introduce errors whose effect should be investigated in future studies.

In conclusion, we present, for the first time, a UM‐EEG as a comprehensive, highly semantic, and confined embedding space that captures physiologically meaningful distances and transitions between EEG states. As a unified embedding space, UM‐EEG affords characterization and classification of brain states in a nuanced way across the health‐disease continuum and enhances current diagnostic and prognostic capabilities. The map provides opportunity for further growth and expansion and opens new avenues for personalized patient monitoring and data‐driven clinical decision support.

## Author Contributions

C.M., L.K., and M.B.W. contributed to the conception and design of the study; L.K., C.M., D.K., A.E., M.H., E.A., S.Z., J.J., R.J.T., and M.B.W. contributed to the acquisition and analysis of the data; L.K. and C.M. contributed to drafting the text or preparing the figures.

## Potential Conflicts of Interest

M.B.W. is a co‐founder, scientific advisor, and consultant to Beacon Biosignals and has a personal equity interest in the company. The remaining authors have no competing interests.

## Supporting information


**Data S1.** Supporting Information.

## Data Availability

Data supporting the study is publicly accessible. This includes the PSG data https://doi.org/10.60508/r0t9-5r24, BS data https://doi.org/10.60508/yvna-zd74, awake routine EEG data https://doi.org/10.60508/g6m4-bf96, cardiac arrest data https://doi.org/10.60508/xwye-v214.
