# Supplementary material for: Toward a Universal Map of EEG: A Semantic, Low‐Dimensional Manifold for EEG Classification, Clustering, and Prognostication
Source: Ann Neurol. 2025 Jun 20;98(2):357–68. doi: 10.1002/ana.27260 (PMC12278028; doi:10.1002/ana.27260)
Supplement: Supplementary file 1 — Data S1. Supporting Information. [file ANA-98-357-s001.doc]

**Supplementary Material**

**Datasets**

To create a universal map of EEG we used data from multiple sources covering a broad range of physiological and pathological states: a sleep dataset from healthy subjects during polysomnography (PSG), a dataset covering the ictal-interictal-injury continuum (IIIC),^1^ data from awake routine EEG recordings,^2^ and burst suppression EEG data.^3,4^ We furthermore use continuous EEG data from patients diagnosed with a disorder of consciousness following cardiac arrest monitored over extended periods of time.^5–7^ Retrospective analysis of data for this project was conducted with waiver of informed consent under approved IRB protocols (BIDMC: 2022P000417; MGH: 2013P001024).

**Polysomnography Data**

The Human Sleep Project (HSP)^8^ dataset consists of clinical polysomnography (PSG) recordings of 25,941 patients, of which 3,609 patients were identified as medically healthy and included in our study.^9,10^ The PSG recordings included six channels of EEG (F3-M2, F4-M1, C3-M2, C4-M1, O1-M2, and O2-M1) based on the international 10-20 system. The dataset includes annotations of wake resting state and sleep stages: wakefulness (W), non-REM sleep stage 1-3 (N1-N3), rapid eye movement sleep (REM). Sleep stages were annotated in 30-second intervals by sleep technologists according to the American Academy of Sleep Medicine (AASM) manual for sleep scoring.

**Ictal-Interictal-Injury Continuum Data**

The Ictal-Interictal-Injury Continuum (IIIC) dataset includes EEG from 1,557 patients, recorded at Massachusetts General Hospital during clinical care.^1^ EEGs were referenced to a bipolar montage including the following channels: Fp1 - F7, Fp1 - F3, F7 - T3, F3 - C3, T3 - T5, C3 - P3, T5 - O1, P3 - O1, Fp2 - F8, Fp2 - F4, F8 - T4, F4 - C4, T4 - T6, C4 - P4, T6 - O2, P4 - O2 and were sampled at 200 Hz. In our study we included 58,117 10-second segments that had received at least 10 independent annotations from a group of 20 experts.^11^ Segments were categorized into the following groups: seizures (SZ), lateralized and generalized periodic discharges (LPD, GPD), lateralized and generalized rhythmic delta activity (LRDA, GRDA). We excluded the group ‘other’ (OTH, including all non-IIIC patterns) from further analysis. The labelled segments were pre-processed by applying a notch filter at 60 Hz and a band-pass filter between 0.5 and 40 Hz.

**Awake Routine EEG Data**

Awake resting-state EEG data was extracted from the Harvard Electroencephalography Database (HED) which encompasses data gathered from four hospitals: Massachusetts General Hospital (MGH), Brigham and Women's Hospital (BWH), Beth Israel Deaconess Medical Center (BIDMC), and Boston Children's Hospital (BCH).^2^ The database includes 164,707 EEG studies conducted on 65,167 patients. For our study, we selected 2,355 patients from the database with normal routine EEGs (19 EEG channels, 10-20 system). The purpose of including this dataset was to calibrate differences between the polysomnography and IIIC datasets when training our model. As the datasets originate from different sources with distinct electrode montages, recording devices and pathologies, we aimed to ensure that the model did not merely learn the differences in monitoring setups. The IIIC dataset is a subset of the HED, which also includes healthy resting state EEG data similar to that in the HSP dataset. To calibrate potential differences between the HSP and IIIC datasets, we extracted wake data from both the HSP and the HED and trained our model with wake data from both datasets under the same label (W).

**Burst Suppression Data**

The burst suppression dataset^3,4^ contains EEGs from 20 critically ill neurological patients from the ICU at Massachusetts General Hospital, recorded between August 2010 and March 2012. The recording durations are less than 90 minutes and consist of 19-channel EEGs according to the international 10-20 system. Two experienced clinical electroencephalographers manually annotated these EEGs as either burst or suppression. Definite burst or suppression epochs were defined as segments of the EEG where both reviewers agreed on the classification. Reviewers marked the beginning and end of all instances of “suppressions,” with all remaining EEG segments classified as non-suppressions or bursts.

**Cardiac Arrest Data**

We investigated long-term EEG data from 605 patients with disorders of consciousness due to cardiac arrest from seven academic hospitals in the U.S. and Europe within the International Cardiac Arrest REsearch consortium (I-CARE).^5–7^ These patients were admitted to the ICU after having a cardiac arrest with a return of heat function while remaining in a comatose state. EEG monitoring typically began within hours of cardiac arrest and continued for several hours to days, varying based on each patient's condition. A continuous 19-channel EEG (10-20 system) was applied to all patients, although different channels are available for patients from different hospitals. For all patients the EEG recording start time is provided in hours after the cardiac arrest. Neurological outcomes were assessed 3 to 6 months after cardiac arrest. A good neurological outcome was defined as a Cerebral Performance Category (CPC) score of 1 or 2, indicating minimal to moderate neurologic disability. A poor outcome was defined as a CPC score of 3-5, indicating severe neurologic disability, persistent coma or vegetative state, or death. In this study, we only included patients with a good outcome of CPC 1 or 2 and a poor outcome of CPC 5 (in total 575 patients) as patients with outcomes 3 or 4 usually had large gaps between the end of the EEG recording and their final assessments.

**Data Preprocessing**

We performed the following preprocessing steps to unify the different datasets. First, all data were resampled to 200 Hz, if necessary. A notch filter at 60 Hz was applied to remove line noise along with a 4^th^ order butterworth bandpass filter between 0.5 and 40 Hz. Next, all datasets were re-referenced to a common electrode configuration. The HSP dataset contained six EEG channels: F3-M2, F4-M1, C3-M2, C4-M1, O1-M2, and O2-M1, while the IIIC segments were already re-referenced as a bipolar montage (Fp1 - F7, Fp1 - F3, F7 - T3, F3 - C3, T3 - T5, C3 - P3, T5 - O1, P3 - O1, Fp2 - F8, Fp2 - F4, F8 - T4, F4 - C4, T4 - T6, C4 - P4, T6 - O2, P4 - O2). To unify across datasets, all EEGs were re-referenced to the common configuration: F3 - C3, C3 - O1, F4 - C4, C4 - O2. Since the IIIC dataset had been segmented into 10-second intervals already, non-overlapping 10-second segments were extracted from all other datasets as well. For the sleep data, annotations were provided for 30-second increments of which we extracted the middle 10 seconds.

The IIIC dataset comprised an average of 1,557 patients, with approximately 58,117 segments per class (SZ, GPD, LPD, GRDA, LRDA). For the sleep data, we utilized EEGs from 3,609 patients. To maintain a comparable magnitude of segments to the IIIC classes, we employed a systematic sampling approach. We first determined the number of available segments for each patient across all sleep stages and the wake state. Subsequently, we extracted either the maximum number of available segments or up to 10 segments per class, whichever was lower. This method ensured a balanced representation across sleep stages while preventing oversampling from any single patient or state. For the routine EEG data, 10 segments (each 10-seconds long) were randomly chosen and extracted per patient. For the burst suppression data, 10-second segments in which both experts annotated at least 50% of the time as suppression periods were selected, with a maximum of 2,000 segments per patient or as many as possible, if less than 2,000 segments were available. Due to the limited number of patients (20 patient EEGs), more segments were extracted for each patient here, compared to the other datasets. For the cardiac arrest data, segments were extracted in consecutive, non-overlapping 10-second steps across the full recording duration.

Finally, each 10-second segment from every EEG channel’s signal was converted to a spectrogram (multitaper method^12^ with time bandwidth = 2, number of tapers = 3, window parameters = [0.5, 0.1] min, nfft = 512, detrend option = constant, multiprocess = True, weighting = ‘unity’) resulting in four spectrograms per each 10-second data segment.

**Electrode Configurations IIIC Data**

**Supplementary Figure 1 Map of IIIC data with different electrode configurations.** **(A)** Illustration of EEG channels of IIIC data selected for training. The electrode configuration with 4 channels (light blue) represents the setup used for creating UM-EEG. **(B)** F1 score after training the model for the different electrode configurations on IIIC data. We performed an ANOVA test on the F1 values of the different electrode configurations (p-value: 0.46). The grey plot indicates F1 scores for an SVM trained on our test embeddings with shuffled labels (chance).

**Burst-Suppression Example Patterns**

**Supplementary Figure 2 Sub-analysis of ambiguous or mixed EEG patterns with burst-suppression as an example.** EEG patterns from complete (Burst Suppression Ratio (BSR) 0) to no suppression (BSR 1) are aligned along a continuum space in the map that accurately reflects the BSR gradient.

**Cardiac Arrest Outcome Prediction for each Institution**

**Supplementary Figure 3 Outcome prediction after cardiac arrest where each of the five centers constitutes an independent, out-of-sample validation data set. (A)** ROC curves obtained from class counts. **(B)** ROC curves obtained from coordinate counts. **(C)** Number of patients and AUC-values for each center individually.

**Supplementary Table 1 Parameters for time series analysis.** Symbols denote: a – wake, b - N1, c - N2, d - N3, e – REM, f – SZ, g – LPD, h – GPD, I – LRDA, j – GRDA, k – BS. Words consist of three symbols. Consequently, a word could, for example, be: "aab" or "kak". From these words, we obtain a new time series, e.g. aab kak dff eee eff. All word parameters are then calculated on this time series.

| fwshannon | This parameter takes the histogram of words for a patient’s whole recordings, and calculates the shannon entropy (= content of information) for them |
| --- | --- |
| forbword | Number of words that occur less than 0.01% of the time |
| healthyword | fraction of words that only contain healthy symbols (a, b, c, d, e) |
| deltaword | fraction of words that only consist of the symbol I and j |
| dischargeword | fraction of words that only consist of the symbols g and h |
| bsword | fraction of words that only consist of the symbols g and h |
| sleepword | fraction of words that only consist of the symbols b, c, d, e |
| deepsleepword | fraction of words that only cosist of the symbol d |
| remsleepword | fraction of words that only consist of the symbol e |
| entropy_of_healthy_states | Shannon entropy of the symbol distribution for healthy symbols (a, b, c, d, e) |
| entropy_of_unhealthy_states | Shannon entropy of the symbol distribution for pathological symbols (f, g, h, I , j, k) |
| pathological_to_total | fraction of symbols that are pathological |
| healthy_to_total | fraction of symbols that are healthy (redundant, 1 - pathological) |
| longest_run_of_a | the longest repetition of the symbol a |
| longest_run_of_b | the longest repetition of the symbol b |
| longest_run_of_c | the longest repetition of the symbol c |
| longest_run_of_d | the longest repetition of the symbol d |
| longest_run_of_e | the longest repetition of the symbol e |
| longest_run_of_f | the longest repetition of the symbol f |
| longest_run_of_g | the longest repetition of the symbol g |
| longest_run_of_h | the longest repetition of the symbol h |
| longest_run_of_i | the longest repetition of the symbol i |
| longest_run_of_j | the longest repetition of the symbol j |
| longest_run_of_k | the longest repetition of the symbol k |
| fraction_of_a | Fraction of symbol a |
| fraction_of_b | Fraction of symbol b |
| fraction_of_c | Fraction of symbol c |
| fraction_of_d | Fraction of symbol d |
| fraction_of_e | Fraction of symbol e |
| fraction_of_f | Fraction of symbol f |
| fraction_of_g | Fraction of symbol g |
| fraction_of_h | Fraction of symbol h |
| fraction_of_i | Fraction of symbol I |
| fraction_of_j | Fraction of symbol j |
| fraction_of_k | Fraction of symbol k |
| self_transition_probability | The probability of the transition x -> x |
| lempel_ziv_complexity | Lempel ziv complexity of symbol series |
| recurrence_rate | Recurrence rate of symbol series |
| trapping_time | trapping time of symbol series |
| kolmogorov_complexity | Kolmogorov complexity of symbol series |
| average_symbol_duration | average duration for a symbol to occur in a row |
| symbol_frequency_variability | standard deviation of the symbol frequency distribution |
| symbol_transition_entropy | The entropy of the series of symbol transitions |
| distance_to_healthy | The mean distance of the current 128D point to the healthy centroid |
| angles_to_healthy | The mean current angle (X_(t-1) - X_(t)) to the vector pointing directly to the healthy centroid |

**References**

1. Jing J, Ge W, Hong S, et al. Development of Expert-Level Classification of Seizures and Rhythmic and Periodic Patterns During EEG Interpretation. *Neurology*. 2023;100(17):e1750-e1762. doi:10.1212/WNL.0000000000207127

2. Zafar S, Loddenkemper T, Lee JW, et al. Harvard Electroencephalography Database. doi:10.60508/G6M4-BF96

3. Brandon Westover M, Shafi MM, Ching S, et al. Real-time segmentation of burst suppression patterns in critical care EEG monitoring. *J Neurosci Methods*. 2013;219(1):131-141. doi:10.1016/j.jneumeth.2013.07.003

4. Shafi M, Moura Junior V, Gupta A, Ghanta M, Westover MB. Real-Time Segmentation of Burst Suppression Patterns in Critical Care EEG Monitoring. doi:10.60508/YVNA-ZD74

5. Amorim E, Zheng WL, Lee JW, et al. I-CARE: International Cardiac Arrest REsearch consortium Database. doi:10.13026/M33R-BJ81

6. Amorim E, Zheng WL, Ghassemi MM, et al. The International Cardiac Arrest Research Consortium Electroencephalography Database. *Crit Care Med*. 2023;51(12):1802. doi:10.1097/CCM.0000000000006074

7. Goldberger AL, Amaral LA, Glass L, et al. PhysioBank, PhysioToolkit, and PhysioNet: components of a new research resource for complex physiologic signals. *Circulation*. 2000;101(23):E215-220. doi:10.1161/01.cir.101.23.e215

8. Westover MD PhD MB, Moura Junior V, Thomas R, et al. The Human Sleep Project. doi:10.60508/R0T9-5R24

9. Sun H, Paixao L, Oliva JT, et al. Brain age from the electroencephalogram of sleep. *Neurobiol Aging*. 2019;74:112-120. doi:10.1016/j.neurobiolaging.2018.10.016

10. Paixao L, Sikka P, Sun H, et al. Excess brain age in the sleep electroencephalogram predicts reduced life expectancy. *Neurobiol Aging*. 2020;88:150-155. doi:10.1016/j.neurobiolaging.2019.12.015

11. Jing J, Ge W, Struck AF, et al. Interrater Reliability of Expert Electroencephalographers Identifying Seizures and Rhythmic and Periodic Patterns in EEGs. *Neurology*. 2023;100(17):E1737-E1749. doi:10.1212/WNL.0000000000201670

12. Prerau MJ, Brown RE, Bianchi MT, Ellenbogen JM, Purdon PL. Sleep Neurophysiological Dynamics Through the Lens of Multitaper Spectral Analysis. *Physiology*. 2017;32(1):60-92. doi:10.1152/physiol.00062.2015
